# Supplementary material for: A self-assembled protein β-helix as a self-contained biofunctional motif
Source: Nat Commun. 2025 May 15;16:4535. doi: 10.1038/s41467-025-59873-1 (PMC12081914; doi:10.1038/s41467-025-59873-1)
Supplement: Supplementary file 1 — Supplementary Information [file 41467_2025_59873_MOESM1_ESM.pdf]

Supplementary Information

A self-assembled protein β-helix as a self-contained biofunctional motif

*Camilla Dondi,[1,2] Javier Garcia-Ruiz,[1,3] Erol Hasan,[1] Stephanie Rey,[1] James E Noble,[1] Alex Hoose,[1] Andrea Briones,[1] Ibolya E Kepiro,[1] Nilofar Faruqui,[1] Purnank Aggarwal,[1] Poonam Ghai,[1] Michael Shaw,[1] Antony T Fry,[1] Antony Maxwell,[1] Bart W Hoogenboom,[2] Christian D Lorenz[3] and Maxim G Ryadnov[1,3,]**

[1]National Physical Laboratory, Hampton Road, Teddington, TW11 0LW, UK

[2]London Centre for Nanotechnology, University College London, London WC1H 0AH, UK

[3]Department of Physics, King's College London, Strand Lane, London, WC2R 2LS, UK

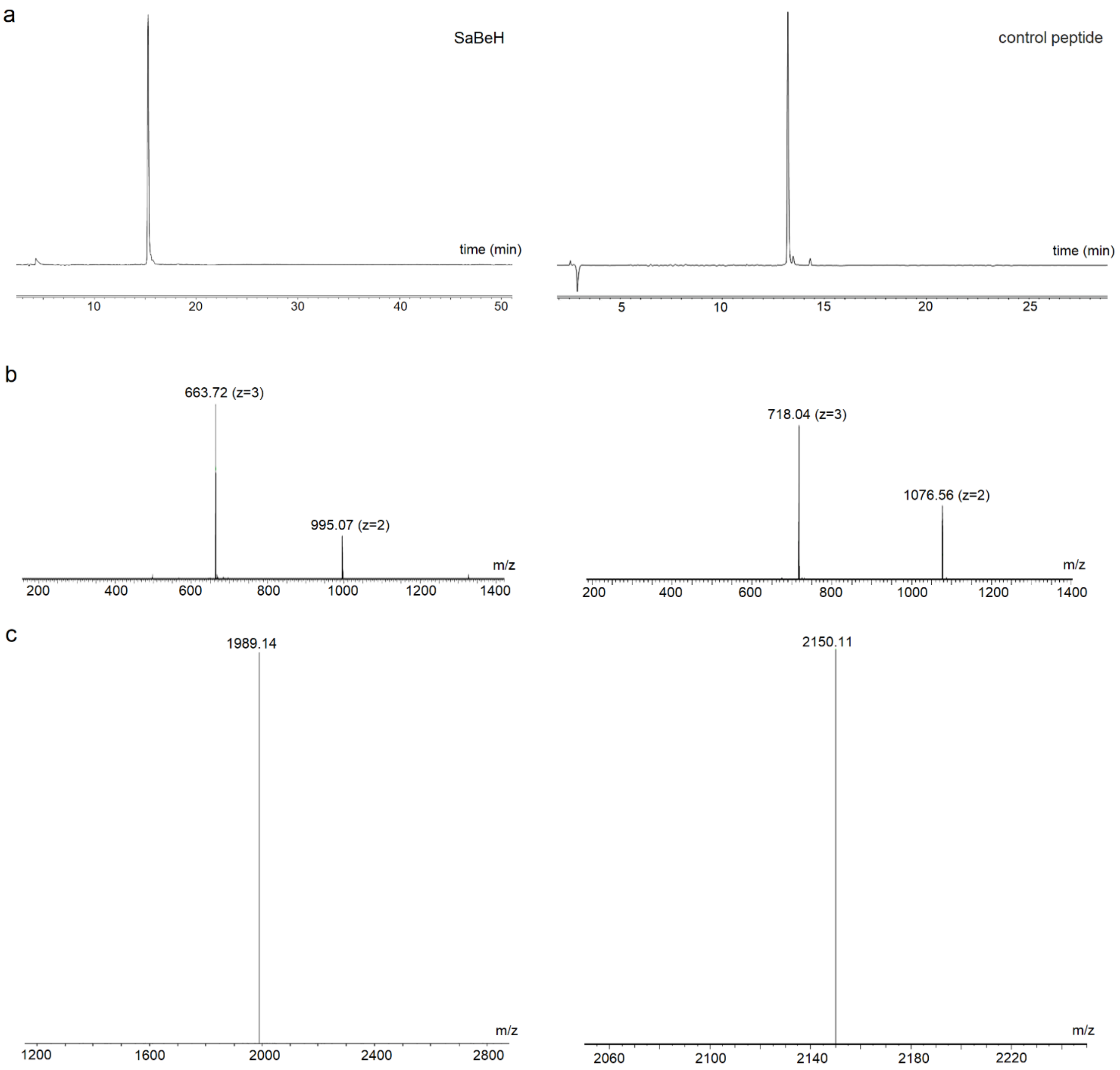

Supplementary Figure 1. Peptide synthesis. a HPLC traces, **b** raw and **c** deconvoluted LC-MS spectra for SaBeH (left) and the non-assembly control peptide (right).

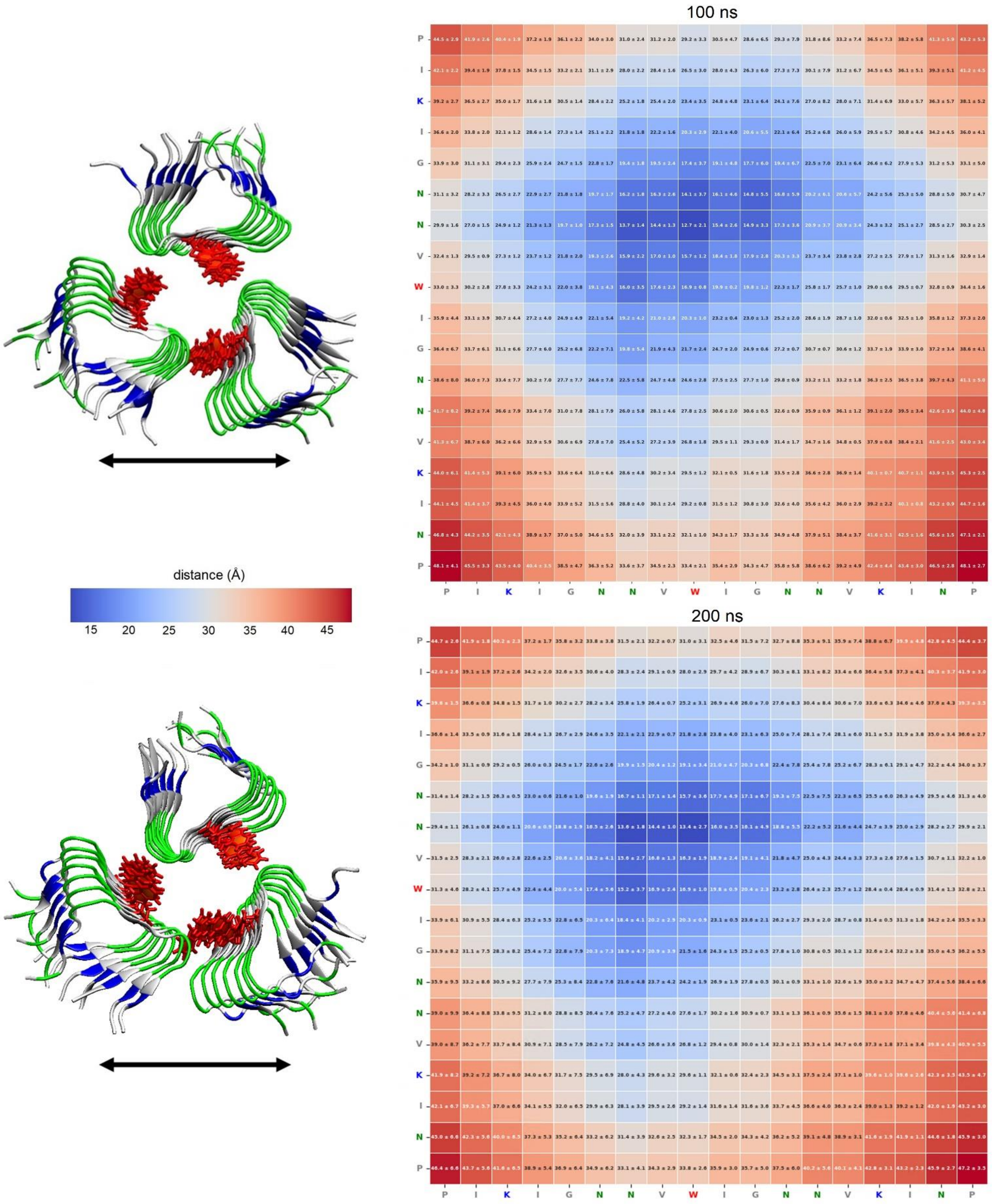

Supplementary Figure 2. Inter-helical interactions. Average distances between Cα atoms of opposite SaBeH helices per β-helical turn after 100-ns MD simulations (upper), with 200-ns MD simulations (lower) given for comparison. Arginine and lysine residues and tryptophan side chains in structures are shown in green, blue, and red, respectively. Every distance is given as a mean with a standard deviation of three replicates.

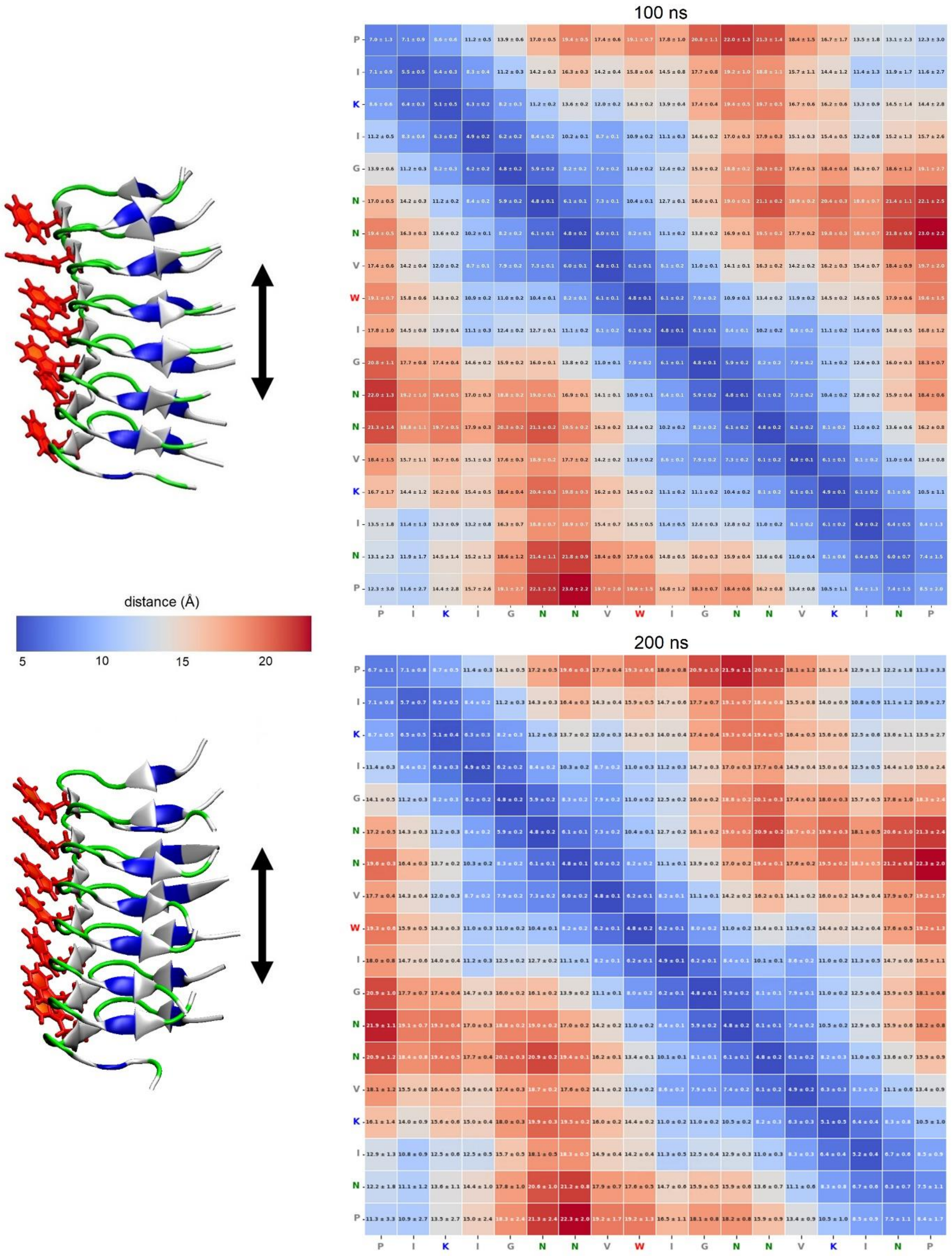

Supplementary Figure 3. Inter-rung interactions. Average distances between Cα atoms of adjacent SaBeH rungs along the β-helix axis after 100-ns MD simulations (upper), with 200-ns MD simulations (lower) given for comparison. Arginine and lysine residues and tryptophan side chains in structures are shown in green, blue, and red, respectively. Every distance is given as a mean with a standard deviation of three replicates.

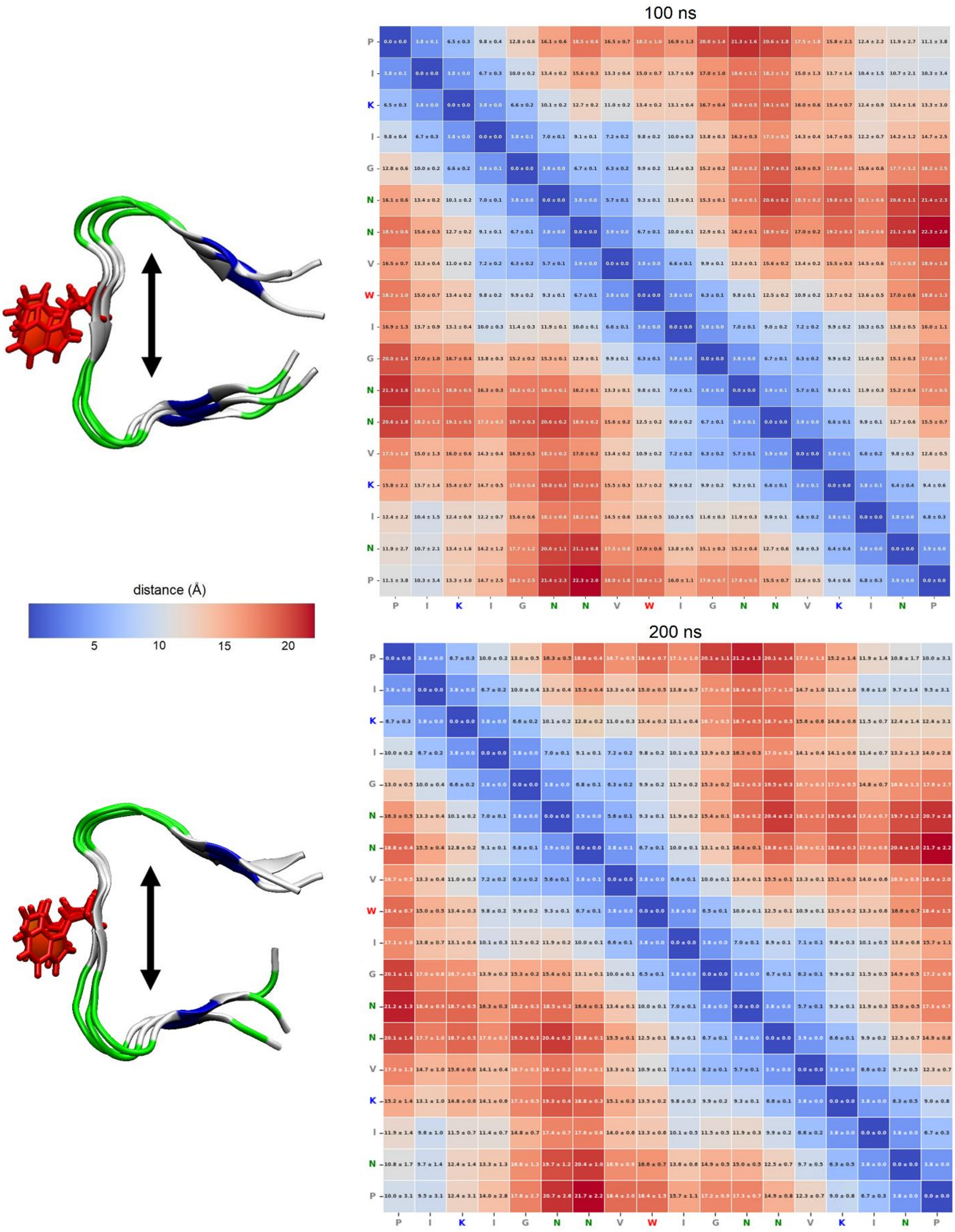

Supplementary Figure 4. Intra-rung interactions. Average distances between Cα atoms within individual SaBeH rungs after 100-ns MD simulations (upper), with 200-ns MD simulations (lower) given for comparison. Arginine and lysine residues and tryptophan side chains in structures are shown in green, blue, and red, respectively. Every distance is given as a mean with a standard deviation of three replicates.

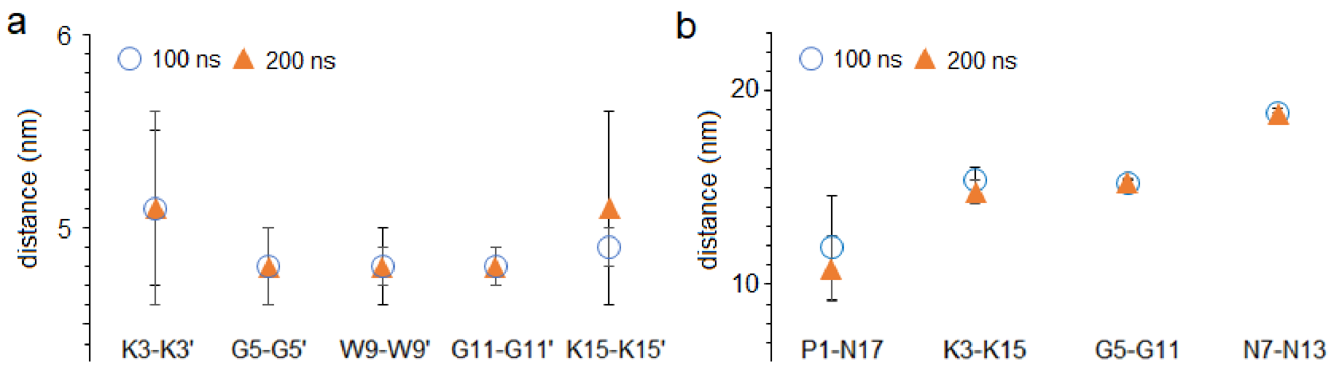

Supplementary Figure 5. Replicate interactions. Average distances between Cα atoms of representative amino-acid pairs for (**a**) adjacent SaBeH rungs along the β-helix axis after 100-ns (open blue circles) and 200-ns (orange triangles) MD simulations and (**b**) within individual SaBeH rungs after 100-ns and 200-ns MD simulations. Every distance is given as a mean with a standard deviation of three replicates. Source data are provided as a Source Data file.

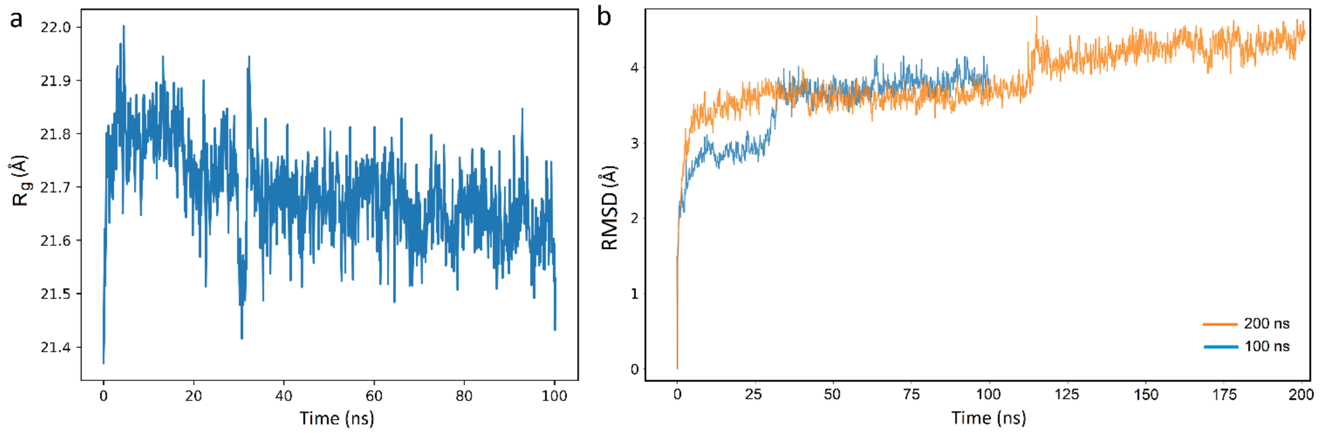

Supplementary Figure 6. Stability of the β-helix assembly. **a** Radius of gyration and **b** RMSD for a seven-turn β-helical trimer simulated over 100 ns (blue) and 200 ns (orange).

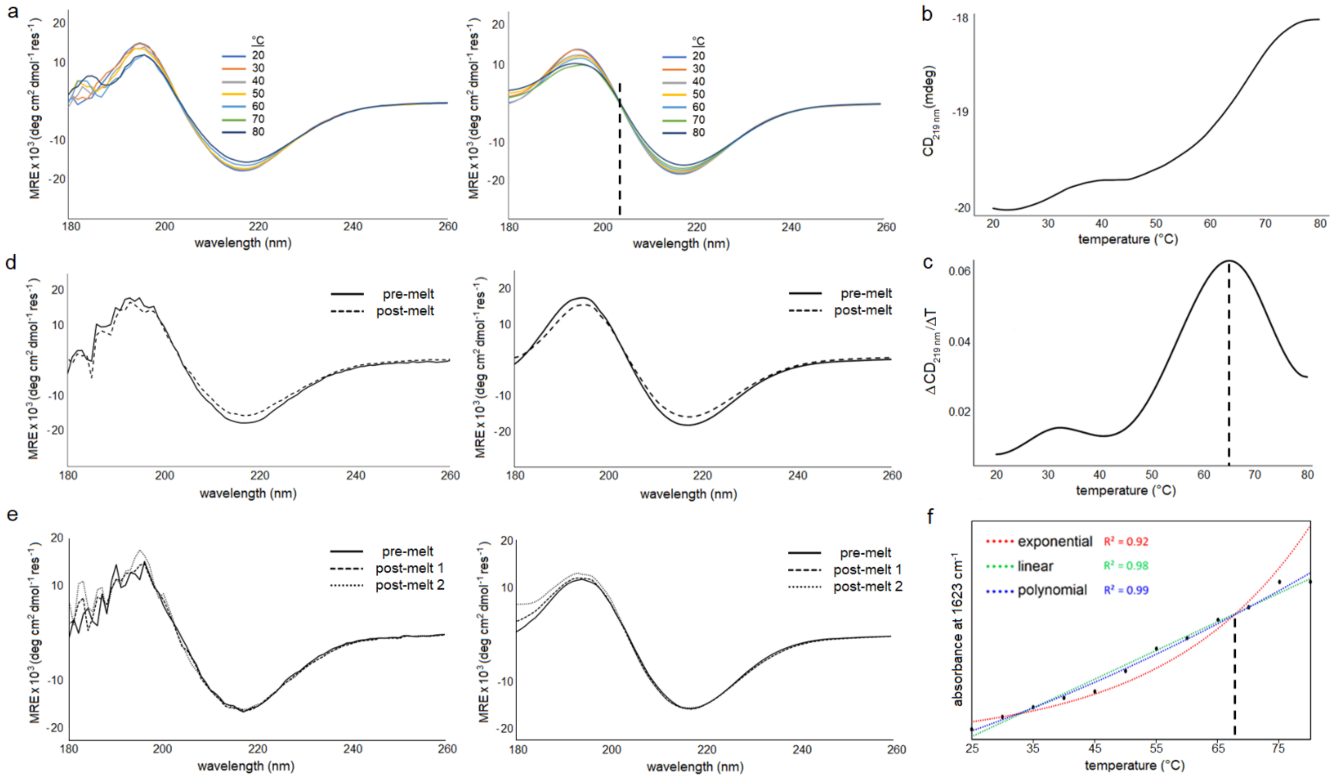

Supplementary Figure 7. β-helix folding. **a** CD spectra, raw (left) and processed using the Savitzky-Golay digital filter (right), recorded every 10 °C during thermal denaturation (20-80 °C). Dashed line indicates the iso-dichroic point at 204 nm. **b** Melting curve recorded at 216 nm. **c** The 1st derivative of the melting curve. Dashed line indicates a transition midpoint at ~65 °C**. d** CD spectra, raw (left) and processed using the Savitzky-Golay digital filter (right), recorded before (solid line) and after (dashed line) the melt. **e** CD spectra, raw (left) and processed using the Savitzky-Golay digital filter (right), recorded before (solid line) and after (dashed line) the first melt and after the second melt (dotted line). **f** Thermal denaturation recorded at 1623 cm^{-1} with individual responses recorded every 5 °C. Dashed line indicates an intersection between linear, exponential and polynomial fits giving a transition midpoint at ~67 °C. Assembly conditions: 60 μM peptide in 10 mM MOPS, pH 7.4, two hours at room temperature.

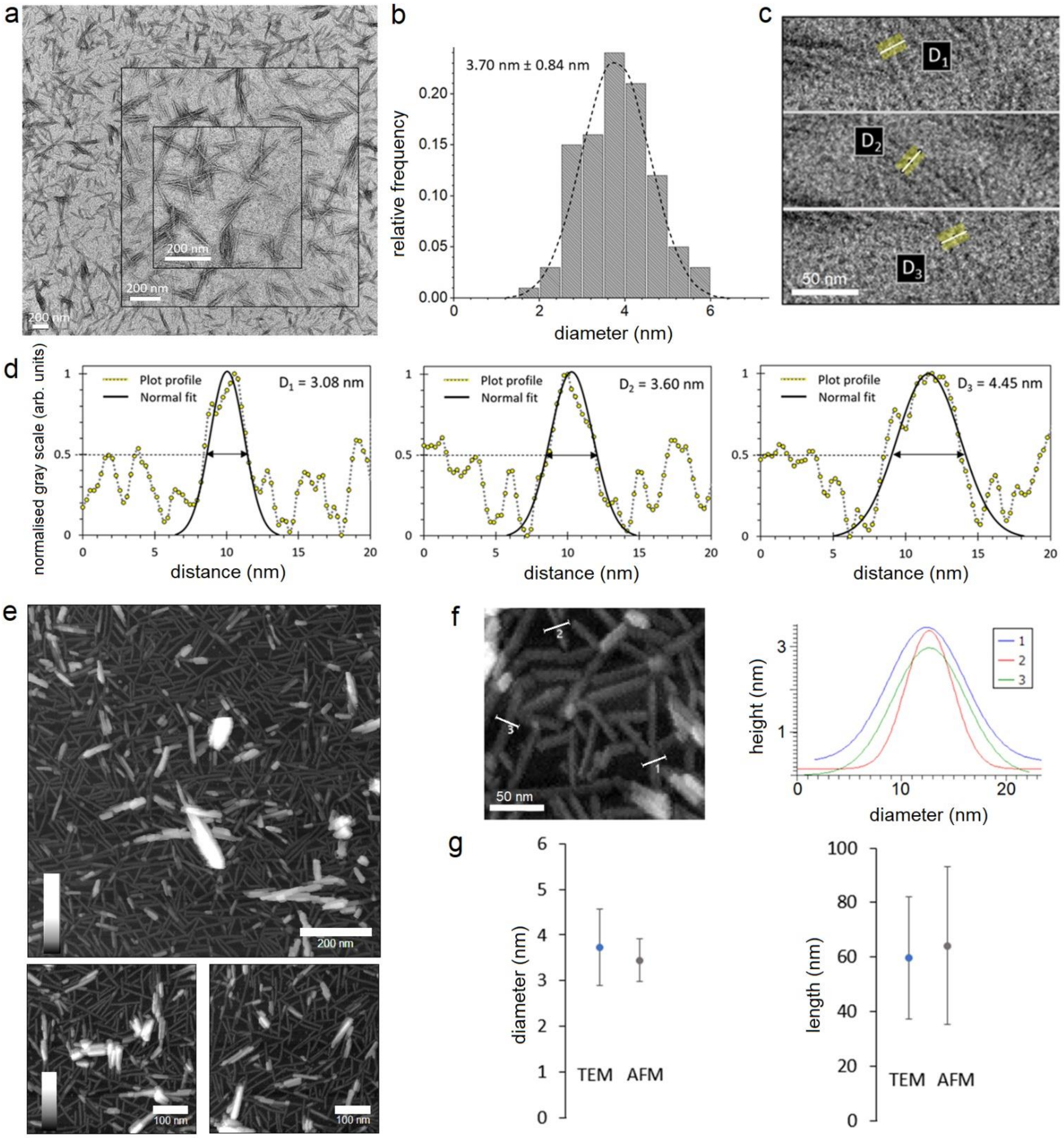

Supplementary Figure 8. β-helix assembly measured by high-resolution microscopy. **a** Electron micrographs of SaBeH cylinders and **b** the distribution of their diameters (full widths at half maximum) with a Gaussian (normal) fit. **c**, **d** Examples of individual diameter measurements by TEM. **e** AFM images of SaBeH cylinders. Colour bars are 80 nm (upper) and 30 nm (lower). **f** Examples of individual diameter measurements by AFM (left) together with their height profiles (right). **g** Average diameters (left) and lengths (right) of SaBeH cylinders determined by TEM and AFM. Assembly conditions: 20-100 µM in 10 mM MOPS, pH 7.4, two hours at room temperature. Images in **a**, **c**, **e, f** are representative of at least three independent experiments. Source data are provided as a Source Data file.

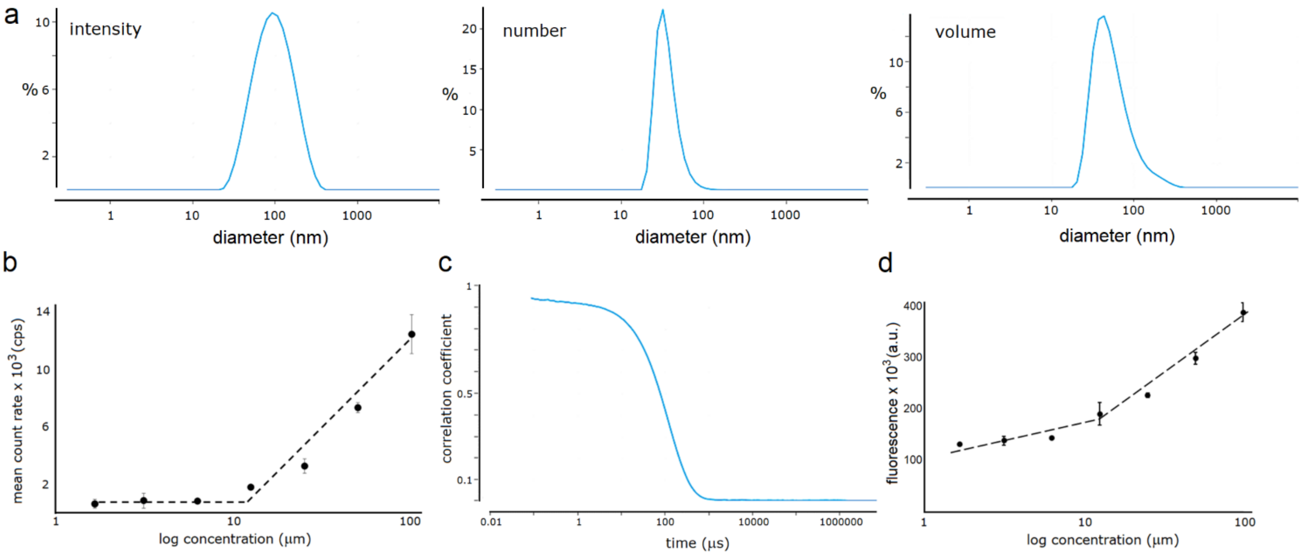

Supplementary Figure 9. β-helix assembly measured by DLS. **a** Size distribution of SaBeH expressed by intensity, number and volume, representative of at least three independent experiments. **b** Critical aggregation concentration (CAC) of SaBeH determined by plotting mean count rates (scattering intensity) with standard deviations versus peptide concentrations for three independent experiments. **c** A representative correlogram of at least three independent experiments with a high intercept at concentrations above CAC. **d** Critical aggregation concentration (CAC) of SaBeH determined by plotting the mean values of fluorescence of thioflavin T (ThT) at 495 nm with standard deviations versus peptide concentrations for three independent experiments. Assembly conditions: 100 µM (unless stated otherwise) in 10 mM MOPS, pH 7.4, 4 h at room temperature.

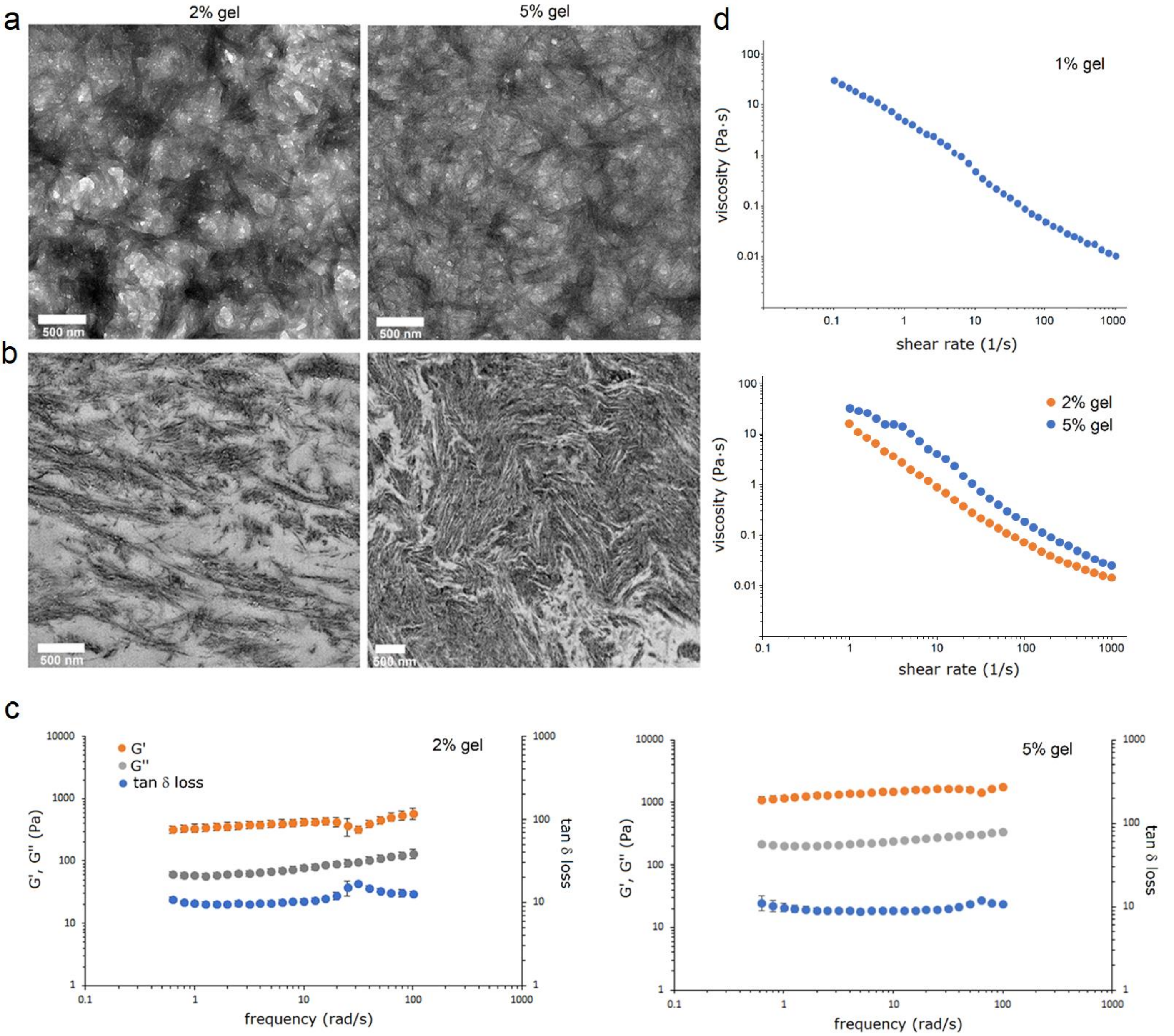

Supplementary Figure 10. β-helix hydrogel measured by microscopy and rheology. Representative electron micrographs of SaBeH hydrogels (2% and 5%, w/v) imaged as is (**a**) and microtomed (**b**). Each image is representative of at least three independent experiments. **c** Storage (G´, orange) and loss (G´´, grey) moduli of the hydrogel and the tangent of the loss angle (tan δ loss, blue) at higher concentrations (2% and 5%, w/v) measured in the 0.5–100 rad/s frequency range. **d** Viscosity of the hydrogel at three different concentrations plotted versus shear rate of 1-1000 1/s. Lower share rates 0.1-1 1/s are also included for 1% hydrogel (w/v), representative of three independent experiments.

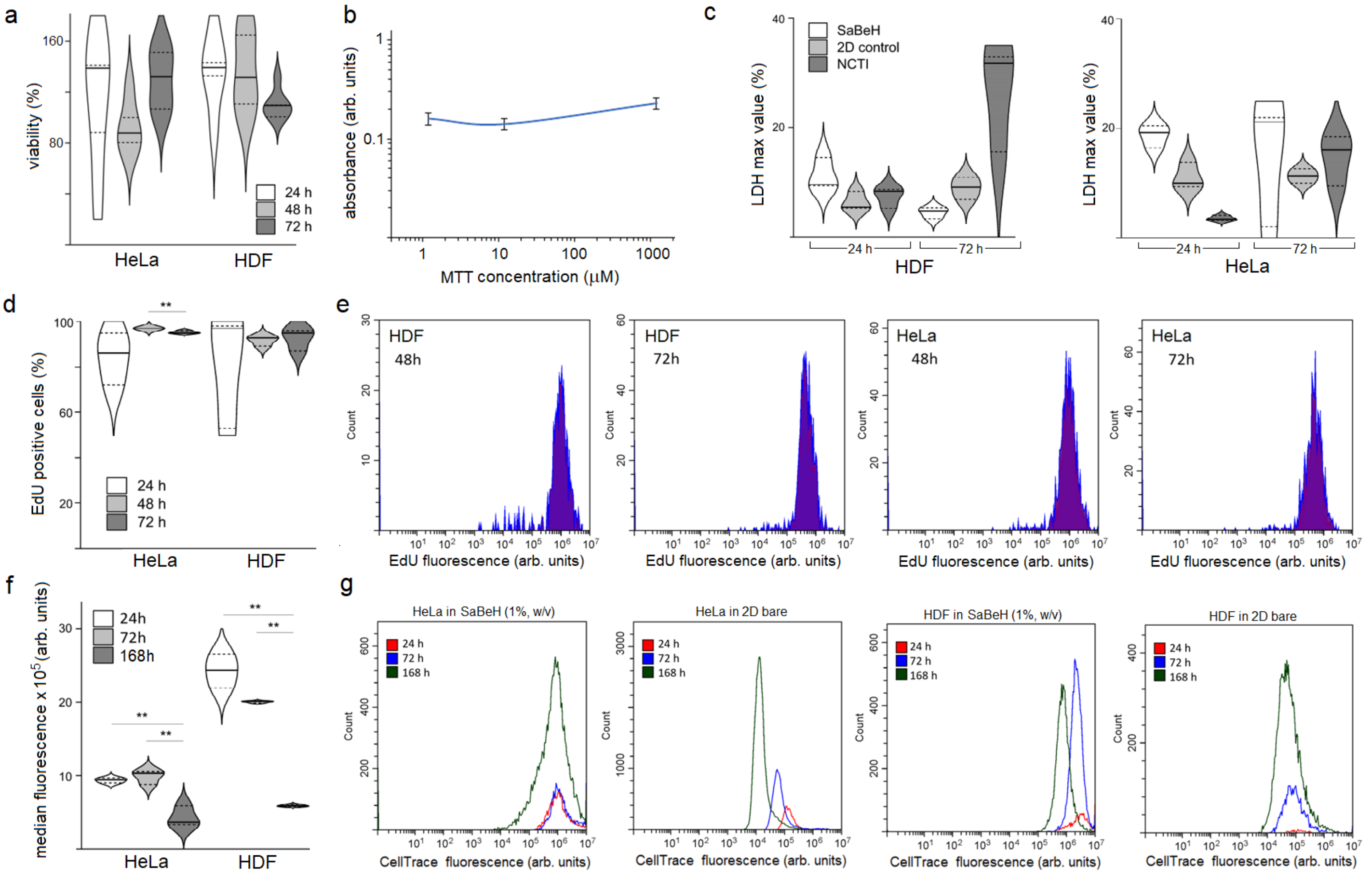

Supplementary Figure 11. β-helix supports 3D cell culture. **a** Cell viability determined by MTT^{TM} assays after subtracting background readouts (bare gels) for cells cultured in SaBeH (1%, w/v) at 24 h (white), 48 h (light grey) and 72 h (dark grey). Total number of metabolically viable cells grown in NCTI was taken as 100%. **b** Absorbance measured at 540 nm versus a concentration log (concentrations) of MTT used in HDFs cultured in SaBeH (1%, v/w) over 24 h. **c** Total LDH released from HDF and HeLa against the total fluorescence of LDH positive control (100%). SaBeH (white), 2D control (light grey), NCTI (dark grey). **d** Total number of EdU-positive cells of the total number of cells at 24 h (white), 48 h (light grey) and 72 h (dark grey). **e** Cell counts versus EdU fluorescence at different time points. Representative flow cytometry charts of EdU-positive cells after cell population gating. At least 10^4 events were gated to extract and analyse cell counts versus fluorescence intensity at 638/660 nm. **f** Median fluorescence of CellTrace-positive cells in SaBeH (1%, w/v) at 24 h (white), 72 h (light grey) and 168 h (dark grey). **g** Cell counts plotted versus CellTrace fluorescence at different time points. Representative flow cytometry charts of CellTrace-positive cells after cell population gating. At least 10^4 events were gated to extract and analyse cell counts versus fluorescence intensity at 405/450 nm. For **a-g** data are presented for at least three independent biological replicates (n = 3). For **a**, **c**, **d**, **f** solid horizontal lines denote median, and upper and lower edges correspond to 75th and 25th percentiles, respectively. Statistical analyses used the analysis of variance (ANOVA) followed by Bonferroni and two-sided t tests, with no significant difference between samples for (**c**) and significantly higher cell numbers for HeLa at 48 h than at 72 h (**d**, $p = 0.029$) and significantly higher median fluorescence at 24 h than 168 h, and at 72 h than 168 h (**f** , HeLa: $p = 0.013$, $p = 0.005$; HDF: $p = 0.005$, $p = 2.27 \times 10^{-8}$). Source data are provided as a Source Data file.

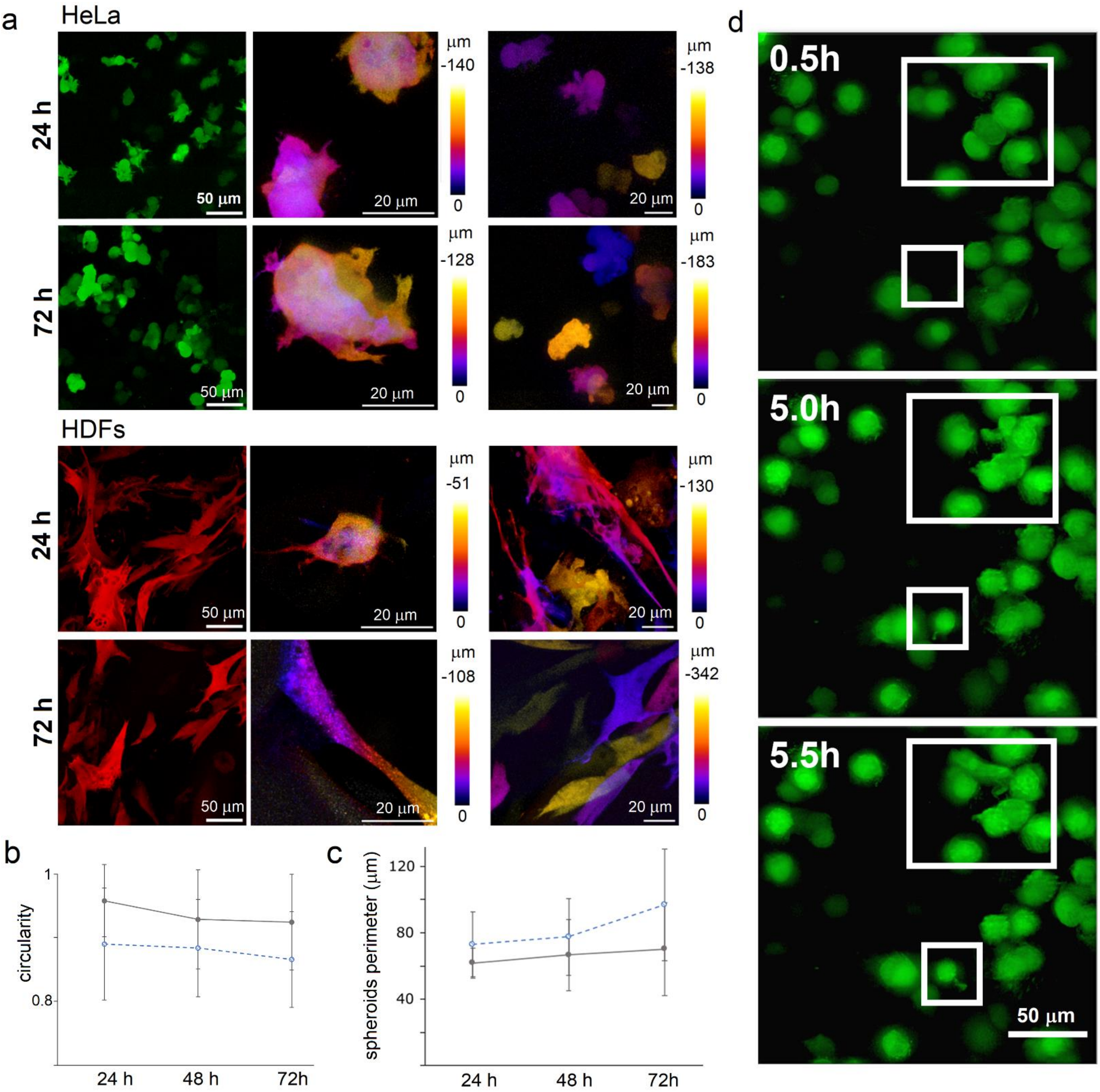

Supplementary Figure 12. β-helix scaffold for 3D cell culture. **a** High magnification fluorescence micrographs of HeLa cells and HDFs, expressing GFP (green) and TurboFP602 (red), respectively. The cells grown in SaBeH and NCTI gels were imaged at fixed time points. Cells located at different depths from the plane of the image are labeled with false colours matching corresponding rainbow scales indicating depth profiles. **b** The circularity of HeLa cells and **c** the perimeter of HeLa spheroids determined in NCTI (grey) and SaBeH (blue) gels using StarDist (https://github.com/stardist/stardist). Data are presented as averages with standard deviations for three independent biological replicates (n=3). **d** Time-lapse fluorescence micrographs of HeLa cells (green) grown in SaBeH gels, representative of at least three independent biological replicates. Source data are provided as a Source Data file.

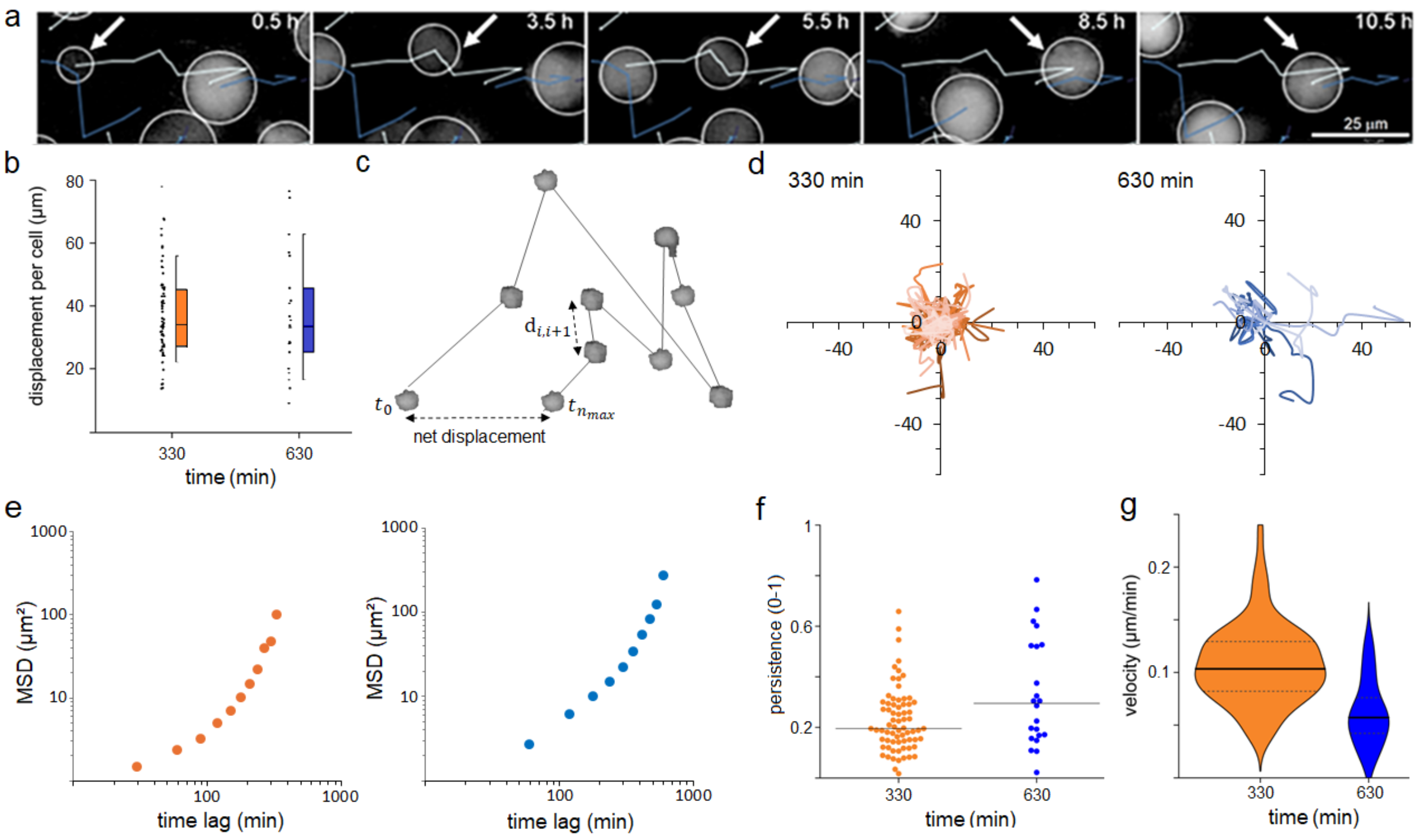

Supplementary Figure 13. β-helix supports cell migration in 3D. **a** Time-lapse micrographs of a single-cell migration trajectory in SaBeH gels (1%, w/v): white arrows follow the trajectory over time (30-630 min). **b** Travel distances (total displacements) of individual HeLa cells (circles) in the gels over the first 330 min (orange) and 630 min (blue). Boxplots: median values (solid horizontal lines), 75th percentile (upper edge), 90th percentile (upper whisker). **c** A cell migration schematic showing a net displacement of a hypothetical cell from starting (0) to final (n_{max}) positions with the total displacement being the sum of individual displacements ($d_{i,\, i+1}$). **d** Cell trajectory maps obtained from **e** ensemble-averaged MSD profiles of individual cells (330 min, n=69; 630 min, n=19). **f** Persistence and **g** velocity of the cells (330 min, n=71; 630 min, n=22) with solid horizontal lines denoting median values, and upper and lower edges of the violin plots corresponding to 75th and 25th percentiles, respectively. Source data are provided as a Source Data file.

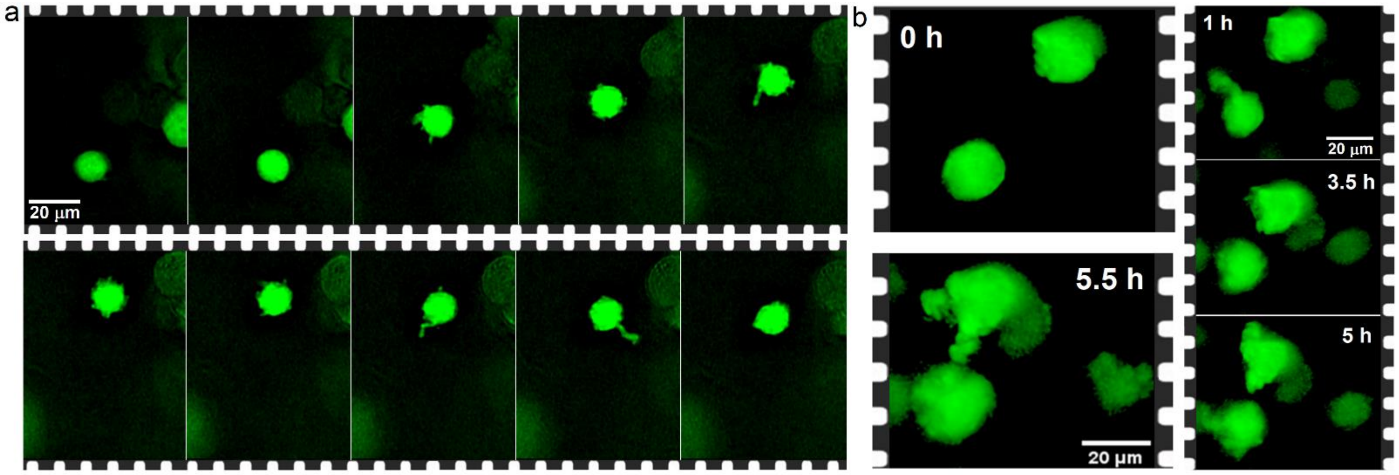

Supplementary Figure 14. β-helix supports cell migration in 3D. Time-lapse micrographs of individual HeLa cells showing **a** continuous filopodia re-arrangements recorded at 30-min intervals, and **b** blebbing dynamics. The images are representative of at least three independent biological replicates.

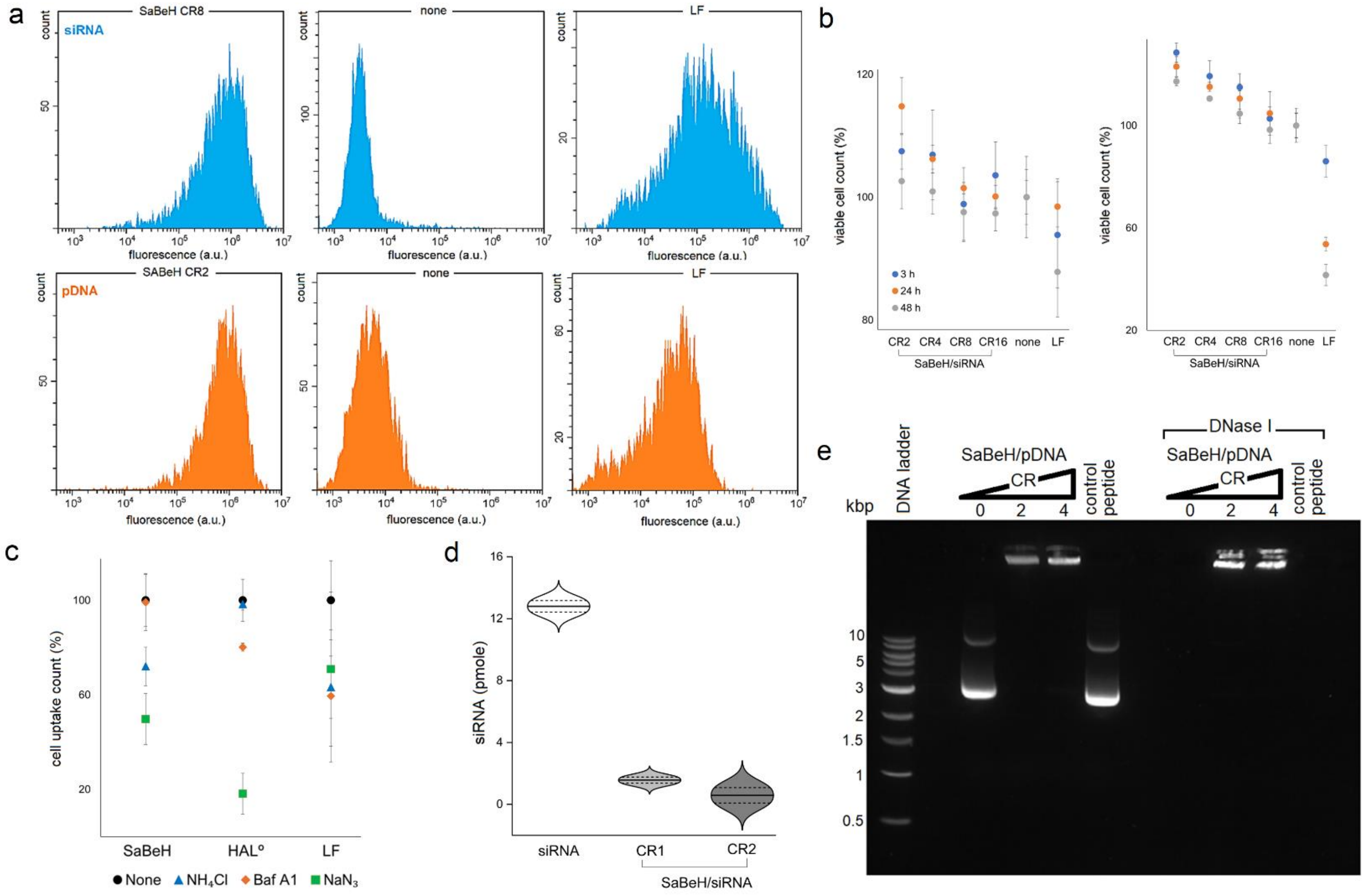

Supplementary Figure 15. Impact of β-helix on intracellular delivery and cell viability. **a** Representative flow cytometry charts of HeLa cells transfected with siRNA labelled with Alexa 647 (blue) and pkm516 pDNA labelled with Cy5 (orange), after cell population gating at 3 hrs post-transfection. At least 10^4 events were gated for each sample to extract and analyse cell counts versus fluorescence intensity at 638/660 nm. **b** Viable cell count for HeLa cells transfected with SaBeH complexed with siRNA and pDNA. The data is expressed as the percentage of viable cells in the total number of cells with standard deviation from experiments performed in triplicate for each peptide-nucleic acid charge ratio. **c** Cell uptake of siRNA by SaBeH (CR4), HaL^{O} (CR4) and LF at 24 hours for HeLa cells pre-treated with cell uptake inhibitors. The uptake is expressed as percentage of median fluorescence (Cy5) recorded for untreated samples, taken as 100%. Data are presented as averages with standard deviations for three independent biological replicates (n=3). **d** Free siRNA from starting total (12.8 pmole) after quantitative encapsulation (11-12 pmole) by SaBeH at CR1-2 showing the mean with standard deviations. Solid horizontal lines denote median values, and upper and lower edges of the violin plots correspond to 75th and 25th percentiles, respectively. **e** 1% Representative agarose gel (1%, w/v) electrophoresis of at least three independent experiments for SaBeH/pDNA at CR1-4 and control peptide mixed with pDNA at CR4 with and without treatment with DNase I. Source data are provided as a Source Data file.

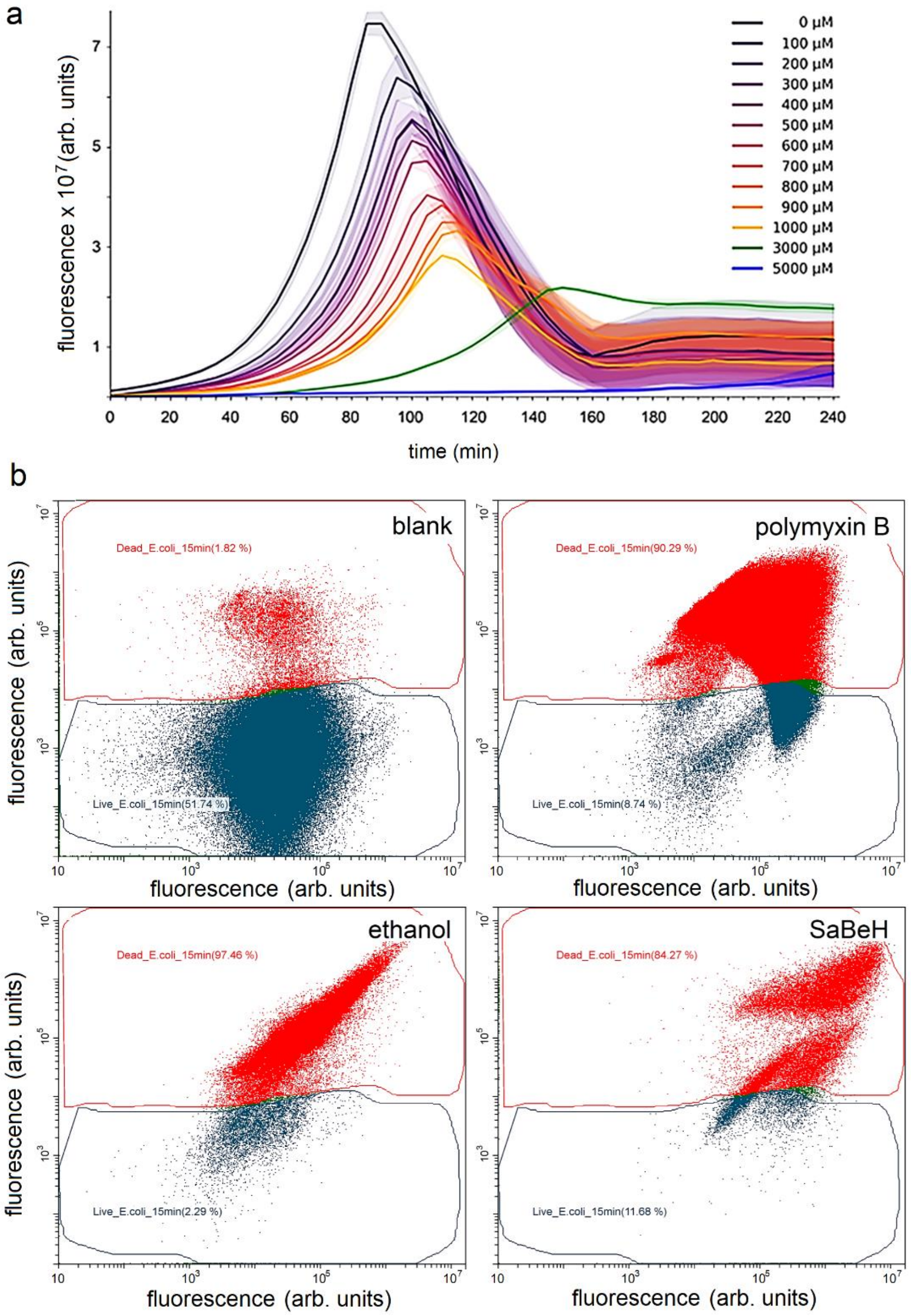

Supplementary Figure 16. Impact of β-helix on bacterial growth. a Growth of *E. coli* determined by PrestoBlue™ assays over first hours of incubation on SaBeH at varied concentrations (100-5000 μM). The concentration-dependent decrease occurs in the time window of the resazurin metabolic conversion – 0-160 min, followed by the depletion of resazurin – 160-240 min. Representative of at least three independent experiments. **b** Representative flow cytometry charts of at least three independent experiments for *E. coli* cells untreated (blank) and treated with polymyxin B, 70% aq. ethanol and SaBeH (100 μM), after cell population gating at 15 min post-treatment. At least 10^5 events were gated for each sample to extract and analyse cell counts. The charts show fluorescence intensity (excitation/emission) at 561/610 nm (Y axis, PI) and 488/525 nm (X axis, SYTO 9).
