# Supplementary material for: A self-assembled protein β-helix as a self-contained biofunctional motif
Source: Nat Commun. 2025 May 15;16:4535. doi: 10.1038/s41467-025-59873-1 (PMC12081914; doi:10.1038/s41467-025-59873-1)
Supplement: Supplementary file 2 — Description of Additional Supplementary Files [file 41467_2025_59873_MOESM2_ESM.pdf]

**Description of Additional Supplementary Files**

**File name:** Supplementary Movie 1
**Description:** Tracking individual cell migration in SaBeH gels (1%, w/v) over the first 10.5 hours.

**File name:** Supplementary Movie 2
**Description:** Tracking individual cell migration in SaBeH gels (1%, w/v) over the first 5.5 hours.
